# Temporal profile of serum orexin-A in neonatal hypoxic-ischemic encephalopathy: a prospective longitudinal study

**DOI:** 10.1186/s12887-026-07727-0

**Published:** 2026-09-21

**Authors:** Pınar Gençpınar, Gunce Basarir, Atilla Ersen, Defne Engür, Nihal Olgaç Dündar, Hüseyin Üstün, Melike Kefeli Demirel, Fatma Ceren Sarıoğlu, Banu İşbilen Başok

**Affiliations:** 1 Department of Pediatric Neurology, Faculty of Medicine, İzmir Katip Çelebi University, İzmir, Türkiye; 2 Department of Pediatric Neurology, İzmir City Hospital, İzmir, Türkiye; 3 Department of Pediatric Neurology, Tepecik Training and Research Hospital, University of Health Sciences, İzmir, Türkiye; 4 Department of Pediatric Neurology, Haseki Training and Research Hospital, University of Health Sciences, Istanbul, Türkiye; 5 Department of Neonatology, Tepecik Training and Research Hospital, University of Health Sciences, İzmir, Türkiye; 6 Department of Radiology, Tepecik Training and Research Hospital, University of Health Sciences, İzmir, Türkiye; 7 Department of Radiology, Faculty of Medicine, Dokuz Eylul University, İzmir, Türkiye; 8 Department of Medical Biochemistry, Tepecik Training and Research Hospital, University of Health Sciences, İzmir, Türkiye; 9 Department of Medical Biochemistry, Dr Behcet Uz Children’s Health and Surgery Training and Research Hospital, University of Health Sciences, İzmir, Türkiye

**Keywords:** Biomarker, Hypoxic-ischemic encephalopathy, Neuroprotection, Newborn, Orexin-A, Therapeutic hypothermia

## Abstract

**Background:**

No serum biomarker has yet been validated for clinical use in neonatal hypoxic-ischemic encephalopathy (HIE). Orexin-A is a hypothalamic neuropeptide that has demonstrated neuroprotective effects in experimental models of cerebral hypoxia–ischemia. We aimed to describe the early temporal profile of serum orexin-A in neonates with HIE, to compare its longitudinal change according to therapeutic hypothermia (TH) exposure, and to examine its relationship with clinical severity, electrophysiological findings and brain imaging.

**Methods:**

This prospective longitudinal observational study enrolled 41 neonates born at ≥ 36 weeks’ gestation in whom HIE was diagnosed within the first 6 postnatal hours. Serum orexin-A was measured at 6–12 h and at 72–96 h after birth. Longitudinal changes were compared between neonates who received TH and those managed without TH, and were examined against clinical severity, amplitude-integrated and video electroencephalography (EEG), brain magnetic resonance imaging (MRI) and routine metabolic indices.

**Results:**

Twenty-two neonates received TH and 19 were managed without TH. Orexin-A concentrations did not differ between groups at either time point (6–12 h, *p* = 0.078; 72–96 h, *p* = 0.082). The within-subject change from 6–12 h to 72–96 h did differ, with relative preservation under TH and a decline without it (median + 31.2 vs. −54.5 pg/mL; *p* = 0.002), and more neonates in the TH group had a higher value at the second measurement (*p* = 0.012). Orexin-A fell significantly over time in the normothermia group (*p* = 0.005). The change also differed by amplitude-integrated EEG background (*p* = 0.010), although abnormal tracings were almost confined to the TH group. Neither concentration nor change was related to video EEG findings, MRI abnormality or metabolic indices. Higher 72–96 h concentrations tracked clinical severity only in outlier-sensitive analyses.

**Conclusions:**

Serum orexin-A followed different early trajectories in neonates managed with and without hypothermia, and the clinical signal lay in the direction of change rather than in any single concentration. Because treatment allocation was inseparable from disease severity, these trajectories cannot be attributed to cooling itself. Orexin-A behaves as a marker of functional state rather than of tissue injury, and serial sampling in larger, severity-matched cohorts with neurodevelopmental follow-up is required before any clinical role can be defined.

**Supplementary Information:**

The online version contains supplementary material available at https://doi.org/10.1186/s12887-026-07727-0.

## Introduction

Neonatal encephalopathy is a disturbance of central nervous system function that usually presents with altered consciousness, hypotonia and seizures [1]. Hypoxic-ischemic encephalopathy (HIE), which follows a reduction in cerebral oxygen delivery and blood flow, is the leading cause of encephalopathy in near-term and term newborns [2] and an important cause of neonatal death and long-term neurodevelopmental disability. Its incidence is approximately 1.7 per 1000 live births in high-income countries and 2.3–30.6 per 1000 in resource-limited settings [3, 4].

HIE arises from a series of physiological, cellular and molecular derangements that follow anoxic brain injury, among them oxidative stress, intracellular calcium accumulation, mitochondrial dysfunction, glutamatergic excitotoxicity, inflammation and apoptosis [5]. Based on pathophysiological changes, HIE can be categorized into four phases. The acute phase covers the first minutes after the hypoxic-ischemic (HI) event, when primary neuronal death follows energy failure, disturbed oxidative metabolism, cytotoxic edema and the accumulation of excitotoxins [6]. The latent phase lasts up to 6 h and corresponds to the timeline during which neuroinflammation and the continuation of activated apoptotic cascades occur [6]. The secondary phase, between 6 and 15 h after the insult, is marked by cytotoxic edema, excitotoxicity and cerebral hyperperfusion [7]. The tertiary phase, extending over the following weeks to months, involves remodeling of the injured brain, astrogliosis and late cell death [8].

The diagnosis of HIE rests on a combination of clinical, neuroimaging and laboratory findings: evidence of an acute hypoxic event, neurological signs, low Apgar scores, cord or arterial blood gas values, hypoxic injury on neuroimaging, and the exclusion of other causes [9]. Severity and prognosis are commonly graded with the modified Sarnat and Thompson scoring systems [10, 11], although both have limitations as diagnostic and prognostic tools. Therapeutic hypothermia (TH), the standard treatment for moderate-to-severe HIE, is initiated on the basis of clinical signs of encephalopathy [12], so infants who only later progress to moderate-to-severe encephalopathy may be missed. There is therefore a need to predict development of encephalopathy, not just to diagnose encephalopathy to extend the therapeutic benefit in HIE. Several candidate biomarkers have been studied, including neuron-specific enolase (NSE), S100B, glial fibrillary acidic protein (GFAP), ubiquitin carboxyl-terminal hydrolase-L1 (UCH-L1) and glutamic-pyruvic transaminase-2 [13], but none has yet proved sufficient for clinical use.

Orexins are neuropeptides that regulate food intake and energy homeostasis, neuroendocrine and autonomic function, thermoregulation, pain perception and the sleep–wake cycle [14, 15]. Orexin-A (hypocretin-1) is neuroprotective in cerebral hypoxia and ischemia [16, 17] and has been proposed as a marker of the progression of ischemic brain damage. In contrast to the structural proteins released when brain tissue is destroyed, orexin-A is a signaling peptide whose circulating concentration should follow the functional state of the orexinergic system; that output is dynamic and stress-responsive, and may therefore be more informative as a trajectory than as a single reading. On this basis we set out to describe the early temporal profile of serum orexin-A in neonates with HIE, to compare its longitudinal change between clinical management groups, and to examine how it relates to disease severity, electrophysiological findings and brain imaging.

## Methods

### Study population

This prospective longitudinal observational study was conducted over 12 months in the neonatal intensive care unit of a tertiary center. Forty-one newborns with HIE were enrolled: 22 received TH (hypothermia group) and 19 did not (normothermia group). The normothermia group comprised infants with mild (modified Sarnat stage I) HIE, who did not meet the criteria for cooling defined below. Infants born at ≥ 36 weeks’ gestation and enrolled within the first six postnatal hours were eligible. Newborns with early-onset neonatal sepsis, intracranial hemorrhage, major congenital anomalies, extreme hyperbilirubinemia or metabolic disturbances such as hypoglycemia were excluded (Supplementary Figure S1).

### Diagnosis of hypoxic-ischemic encephalopathy

HIE was diagnosed within the first six postnatal hours according to the national guideline of the Turkish Neonatal Society [18] and the latest international consensus definitions [2, 9, 19]. Diagnosis required a gestational age of ≥ 36 weeks and evidence of both perinatal hypoxia-ischemia and neonatal encephalopathy. Evidence of an HI event included an Apgar score < 5 at both 5 and 10 min, an umbilical cord blood pH < 7.00, and/or a base deficit > 12 mmol/L. When identifiable, acute peripartum or intrapartum events included uterine rupture, placental abruption, cord prolapse, maternal hypotension or hypoxemia, amniotic fluid embolism, vasa previa, and fetomaternal hemorrhage. A neonatologist and a pediatric neurologist performed the neurological examination within the first six hours of life and graded the encephalopathy according to the modified Sarnat classification as mild (stage I), moderate (stage II), or severe (stage III) [10]. Multiorgan involvement and, subsequently, an imaging pattern typical of HI injury were regarded as supportive.

### Eligibility for therapeutic hypothermia

The indication for TH was also determined using the treatment criteria specified in the Turkish Neonatal Society guideline [18] and the latest framework of the British Association of Perinatal Medicine [12]. TH was initiated in infants born at ≥ 36 weeks’ gestation who were less than six hours old and met both criteria A and B. Criterion A required at least one marker of perinatal hypoxia–ischemia: cord blood or first-hour blood gas pH < 7.00, base deficit ≥ 16 mmol/L, an Apgar score < 5 at 10 min, or continued need for resuscitation at 10 min. Criterion B required moderate or severe encephalopathy, corresponding to modified Sarnat stage II or III [10]. In line with the national guideline, amplitude-integrated EEG was used for diagnostic support and for monitoring and was not applied as an entry criterion for cooling. Infants with mild encephalopathy (stage I) were not eligible for cooling and were managed at normothermia. TH was initiated within six hours of birth and a core temperature of 33.5 ± 0.5 °C was maintained for 72 h, followed by controlled rewarming.

### Study design and sample collection

Disease severity was determined by physical examination, clinical scoring (modified Sarnat and Thompson systems) [10, 11], amplitude-integrated and video electroencephalography (EEG), and brain magnetic resonance imaging (MRI). Using the modified Sarnat classification, infants were graded as having mild (stage I), moderate (stage II) or severe (stage III) encephalopathy [10]. By the Thompson system (maximum 22), a score of 1–10 indicated mild, 11–14 moderate and ≥ 15 severe HIE [11]; the score was recorded on the first, second and third days of life. Amplitude-integrated and video EEG were recorded for at least 24 h beginning within the first 12 h of life; for analysis, the amplitude-integrated EEG background and the video EEG recording were each classified as normal or abnormal. Brain MRI (GE 1.5T Optima MR360) was performed in all patients, and T1, T2 and diffusion-weighted signal abnormalities were evaluated; images were classified as normal or abnormal for analysis.

Venous blood was collected from all participants into plain tubes (BD Vacutainer^®^ SST II Advance, 5 mL; USA) at 6–12 h and 72–96 h. Serum was separated by centrifugation (1,500 g, 10 min) within one hour of sampling, aliquoted, and stored at − 80 °C. Serum orexin-A was measured by quantitative enzyme-linked immunosorbent assay (ELISA) (Elabscience Biotechnology Inc., TX, USA; Catalog E-EL-H1015) per the manufacturer’s instructions. Intra- and inter-assay coefficients of variation were < 4.5% and < 5.3%, respectively; the limit of detection was 37.5 pg/mL and the measurement range 62.5–4000 pg/mL.

### Statistical analysis

Analyses were performed in IBM SPSS v29.0. Categorical variables are reported as frequency (n) and percentage. Because serum orexin-A concentrations and their within-subject changes departed from normality in several key subgroups (Shapiro–Wilk *p* < 0.05), continuous orexin-A variables are summarized as median (interquartile range, IQR) and non-parametric tests were used as the primary approach; normally distributed descriptive variables are given as mean ± standard deviation (SD). Normality was assessed with the Shapiro–Wilk test and homogeneity of variance with Levene’s test. Between-group comparisons used the Mann–Whitney U test, within-group temporal changes the Wilcoxon signed-rank test, comparisons across Sarnat stages the Kruskal–Wallis test, and categorical variables the Fisher exact test. Correlations between orexin-A and Thompson score were assessed with Pearson and Spearman coefficients. Cohen’s d and Cliff’s delta quantified the magnitude and consistency of the primary between-group difference in orexin-A change.

Given the small, severity-imbalanced sample, sensitivity analyses excluded high-leverage observations, a permutation test (20,000 iterations) was applied, and a multivariable linear regression (outcome: change in orexin-A; predictors: management group, baseline orexin-A, gestational age, birth weight) tested whether the group difference was independent of baseline characteristics. Orexin-A at each time point and its change were compared across the dichotomized electrophysiological and imaging categories (amplitude-integrated EEG, video EEG, MRI) and across the presence or absence of seizures with the Mann–Whitney U test, and their correlations with creatine kinase, lactate dehydrogenase, C-reactive protein, pH, base deficit and Thompson scores were assessed with the Spearman rank coefficient. Because abnormal electrophysiological and imaging findings clustered in the TH group, any association identified in this way was re-examined within the TH group alone. Discrimination of management groups by orexin-A change was summarized with the area under the receiver operating characteristic curve (AUC). *P* < 0.05 was considered significant.

## Results

### Patient characteristics

Baseline characteristics are summarized in Table 1. Because eligibility for TH is determined by the severity of encephalopathy, the groups were not severity-matched. The hypothermia group had a lower gestational age (37.8 ± 1.8 vs. 39.1 ± 1.6 weeks, *p* = 0.03), lower birth weight (2.97 ± 0.58 vs. 3.33 ± 0.3 kg, *p* = 0.019), smaller head circumference (*p* = 0.016), higher early Thompson scores (*p* = 0.003) and a greater base deficit (*p* = 0.025); seizures occurred almost exclusively in this group (*p* = 0.004). All 19 normothermia neonates were Sarnat stage I, whereas the TH group included stages I (*n* = 7), II (*n* = 12) and III (*n* = 3) (*p* < 0.001). The groups did not differ in maternal age, mode of delivery, sex, need for resuscitation, or 1st- and 5th-minute Apgar scores (all *p* > 0.05).

**Table 1:** Baseline demographic and clinical characteristics by clinical management group

			Hypothermia group (*n* = 22)	Normothermia group (*n* = 19)	*p* value
Gestational age (w)		Mean ± SD	37.8 ± 1.8	39.1 ± 1.6	0.03*
Birth weight (kg)		Mean ± SD	2.97 ± 0.58	3.33 ± 0.3	0.019*
HC (cm)		Median [IQR]	34 [33–35]	35 [34–36]	0.016*
Sarnat stage I / II / III		*n*	7 / 12 / 3	19 / 0 / 0	< 0.001*
6–12 h Thompson score		Median [IQR]	5 [2–7]	2 [1–3]	0.003*
Base deficit (mmol/L)		Median [IQR]	16.3 [13.8–19.8]	13.1 [12.5–14.8]	0.025*
Seizure frequency		*n* (%)	8 (36.4%)	0 (0%)	0.004*
Apgar score	1st minute	Median [IQR]	4 [2–6]	5 [5–7]	0.07
	5th minute	Median [IQR]	7 [6–8]	8 [7–8]	0.3

Data are mean ± SD or median [IQR]. Comparisons by Student’s t-test, Mann-Whitney U, or Fisher exact test as appropriate. *h* hour, *HC* head circumference, *IQR* interquartile range, *n* number, *SD* standard deviation, *w* weeks. *p < 0.05

### Serum orexin-A levels

**Fig. 1:**
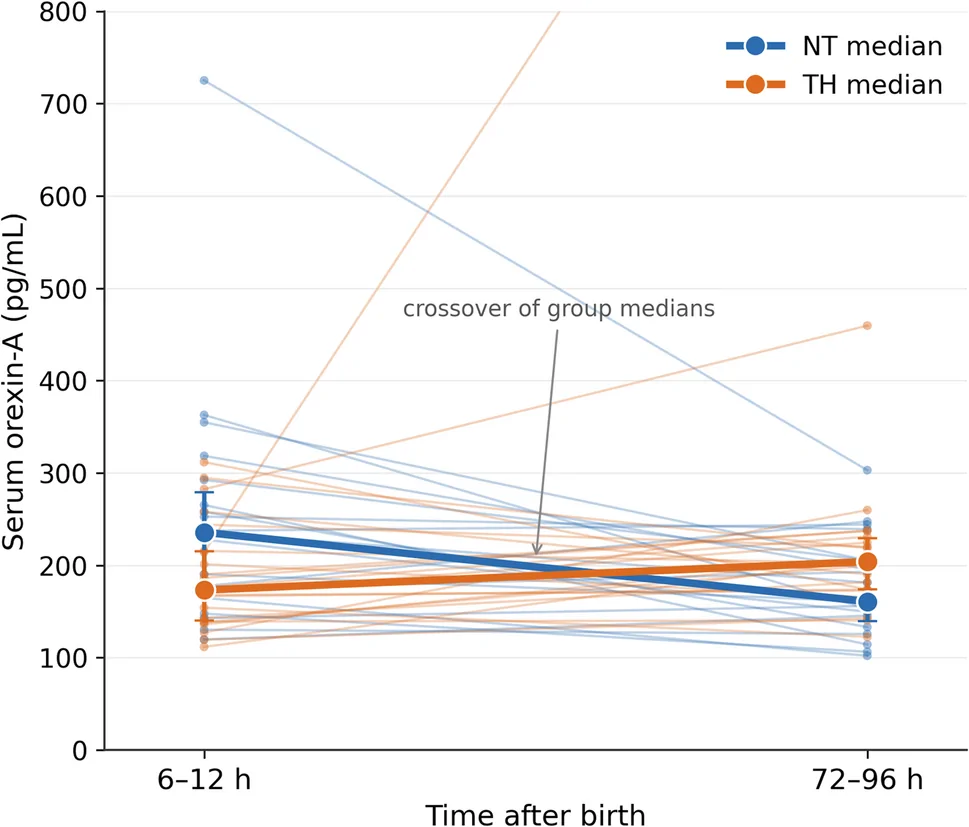
Individual longitudinal trajectories of serum orexin-A from 6–12 h to 72–96 h in each neonate, coloured by clinical management group. h, hour; NT, normothermia; TH, therapeutic hypothermia

**Fig. 2:**
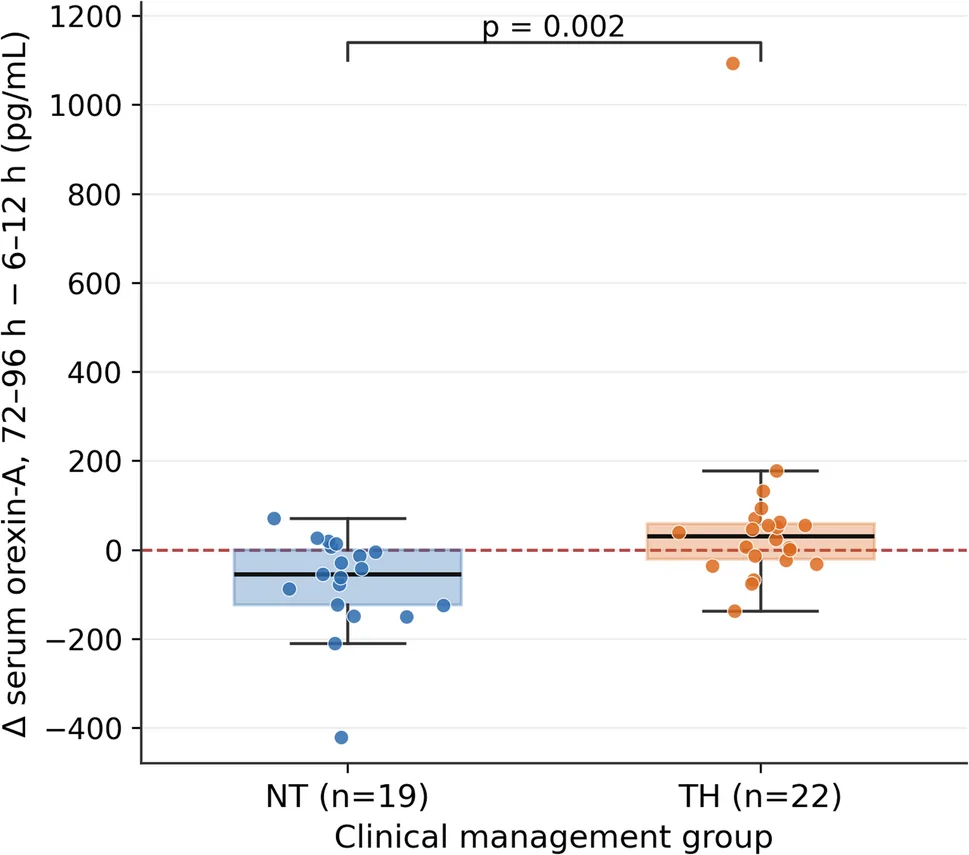
Change in serum orexin-A from 6–12 h to 72–96 h (Δ) by clinical management group. h, hours; NT, normothermia; TH, therapeutic hypothermia

**Fig. 3:**
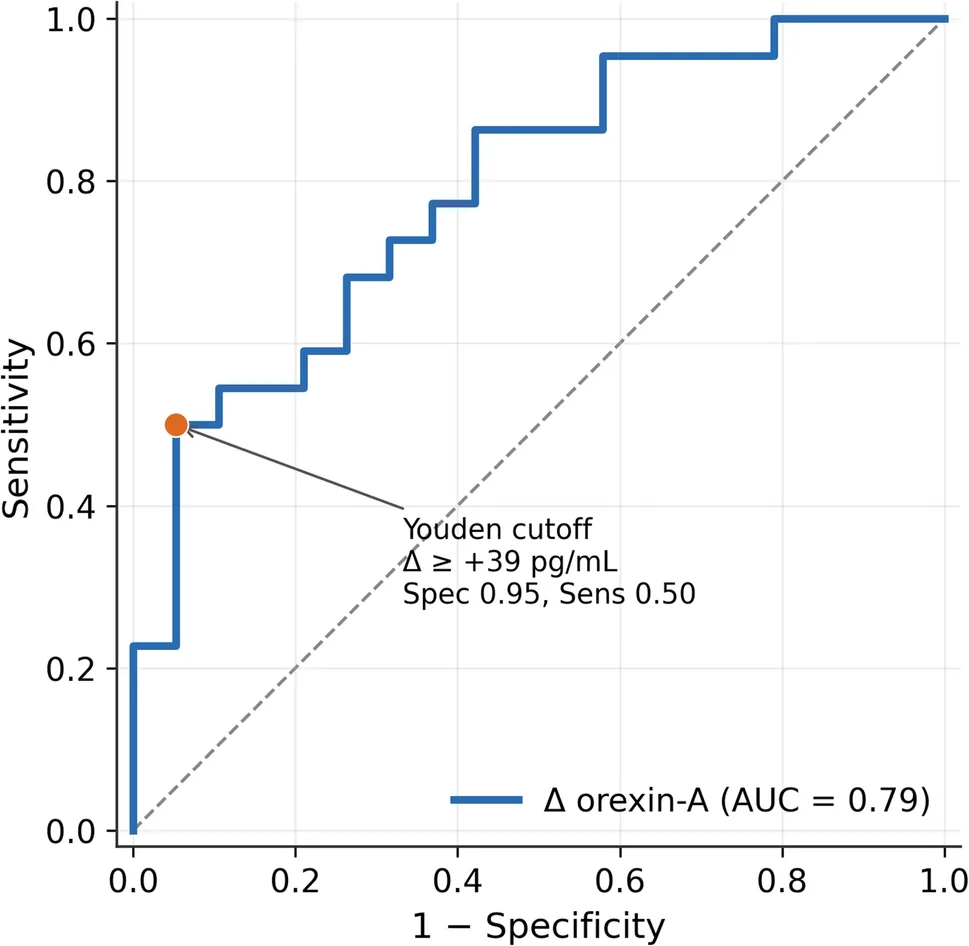
ROC curve for the change in serum orexin-A distinguishing neonates managed with versus without therapeutic hypothermia (AUC = 0.79). AUC, area under the receiver operating characteristic curve; Sens, sensitivity; Spec, specificity

Serum orexin-A did not differ significantly between the hypothermia and normothermia groups at 6–12 h (median 173.2 [140.1–215.2] vs. 235.8 [169.2–278.9] pg/mL, *p* = 0.078) or at 72–96 h (204.0 [173.8–229.4] vs. 160.8 [139.3–205.4] pg/mL, *p* = 0.082). Individual longitudinal trajectories are shown in Fig. 1. The change between the two time points, however, differed between groups, with relative preservation in the hypothermia group and a decline in the normothermia group (median + 31.2 [− 21.2, + 59.9] vs. −54.5 [− 123.8, + 1.0] pg/mL; *p* = 0.002; Welch t-test *p* = 0.017; Fig. 2), corresponding to a medium-to-large effect size (Cohen’s d = 0.75; Cliff’s delta = 0.58). The difference held across sensitivity analyses, remaining significant in a 20,000-iteration permutation test (*p* = 0.001) and after exclusion of the single high-leverage observation (Mann–Whitney *p* = 0.003). Orexin-A change distinguished the two management groups with an AUC of 0.79 (Fig. 3), and the proportion of infants with a higher orexin-A at the second measurement was greater in the hypothermia group (15/22, 68.2% vs. 5/19, 26.3%; *p* = 0.012). Within groups, orexin-A declined significantly in the normothermia group (Wilcoxon *p* = 0.005) but not in the TH group (*p* = 0.113), indicating the absence of the decline seen in milder disease rather than a definite rise (Table 2).

**Table 2:** Serum orexin-A levels and temporal changes by clinical management group

Serum orexin-A	Hypothermia (*n* = 22)	Normothermia (*n* = 19)	*p* value
At 6–12 h (pg/mL)	173.2 [140.1–215.2]	235.8 [169.2–278.9]	0.078
At 72–96 h (pg/mL)	204.0 [173.8–229.4]	160.8 [139.3–205.4]	0.082
Change (72–96 and 6–12 h)	+ 31.2 [− 21.2, + 59.9]	−54.5 [− 123.8, + 1.0]	0.002*
Proportion with increase	15/22 (68.2%)	5/19 (26.3%)	0.012*
Within-group change	*p* = 0.113 (NS)	*p* = 0.005 (decline)	—
Sensitivity analysis of orexin-A change	Permutation *p* = 0.001; outlier-excluded *p* = 0.003	AUC 0.79 (TH vs. NT)	—

Data are median [IQR]. Between-group comparisons by Mann–Whitney U test; proportion comparison by Fisher exact test. Effect size for the change: Cohen’s d = 0.75, Cliff’s delta = 0.58. The between-group difference in change remained significant in a permutation test (20,000 iterations, *p* = 0.001) and after exclusion of the single high-leverage observation (*p* = 0.003). *AUC* area under the receiver operating characteristic curve for distinguishing management groups, *h* hour, *n* number, *NS* not significant, *NT* normothermia, *TH* therapeutic hypothermia. *p < 0.05

At 72–96 h, serum orexin-A was numerically higher in Sarnat stage III neonates on parametric ANOVA (*p* = 0.002), but this rested entirely on a single high-leverage observation (one stage III neonate at 1306.7 pg/mL, against 231.0 and 165.5 pg/mL in the other two). Non-parametric Kruskal–Wallis analysis showed no difference across stages (*p* = 0.38), and the comparison of Sarnat III with stages I and II combined was also non-significant (*p* = 0.24); after exclusion of the high-leverage value, Kruskal–Wallis *p* = 0.74 (Supplementary Table S1). The Pearson correlation between 72–96 h orexin-A and the third-day Thompson score was similarly strong (*r* = 0.72, *p* < 0.001) but outlier-sensitive: Spearman ρ = 0.22 (*p* = 0.16), and after exclusion of the extreme observation Pearson *r* = 0.12 (*p* = 0.46) and Spearman ρ = 0.16 (*p* = 0.33) (Supplementary Table S2). These severity associations are therefore exploratory.

To test whether the between-group difference in orexin-A trajectory was independent of baseline characteristics, a multivariable linear regression was constructed with the change in orexin-A as the outcome and management group, baseline orexin-A, gestational age and birth weight as predictors (Table 3). After adjustment the management-group effect was attenuated and non-significant (β=+55.4 pg/mL, standard error 31.4, *p* = 0.086), the only significant predictor being baseline orexin-A (β=−64.1, *p* = 0.037), a pattern consistent with regression to the mean. The model explained 31.9% of the variance (R²=0.319), indicating that the unadjusted group difference is at least partly attributable to baseline severity.

**Table 3:** Multivariable regression: predictors of change in serum orexin-A

Predictor	β (pg/mL)	SE	t	*p* value
Clinical management group (TH)	+ 55.4	31.4	1.77	0.086
Baseline orexin-A (6–12 h)	−64.1	29.6	−2.17	0.037*
Gestational age (w)	−53.8	31.9	−1.69	0.100
Birth weight (kg)	+ 56.1	31.8	1.76	0.087

Dependent variable: change in orexin-A from 6–12 h to 72–96 h (*n* = 41). Model R²=0.319. The management-group effect loses significance after covariate adjustment, a pattern consistent with confounding by disease severity. *h* hour, *SE* standard error, *TH* therapeutic hypothermia, *w* weeks. *p < 0.05

### Relationship with electrophysiological, neuroimaging, and metabolic findings

Serum orexin-A concentrations at 6–12 h and at 72–96 h were unrelated to the amplitude-integrated EEG background, video EEG findings, brain MRI abnormality and the occurrence of seizures (all *p* > 0.05; Table 4). The change between the two samples differed according to the amplitude-integrated EEG background: infants with an abnormal background showed a median rise of + 23.5 [− 5.8 to + 62.7] pg/mL, whereas those with a normal background showed a median fall of − 36.4 [− 84.9 to + 17.7] pg/mL (*p* = 0.010), and a higher proportion of the abnormal-background group had a rise (11/15, 73.3% vs. 9/26, 34.6%; *p* = 0.025). The difference persisted after removal of the single high-leverage observation (*p* = 0.019). Abnormal backgrounds were, however, almost confined to the hypothermia group (14 of 15), and within that group alone the difference was no longer apparent (*p* = 0.402); the association therefore restates the management-group difference rather than adding to it. No corresponding differences were seen for video EEG (*p* = 0.53), MRI abnormality (*p* = 0.16) or seizures (*p* = 0.36). Neither the 72–96 h concentration nor the change correlated with creatine kinase, lactate dehydrogenase, C-reactive protein, pH or base deficit (all |ρ|≤0.22, *p* ≥ 0.17; Table 4). Among the daily Thompson scores, only the second-day score showed a weak rank correlation with the 72–96 h concentration (ρ = 0.36, *p* = 0.023), which fell to ρ = 0.30 (*p* = 0.057) once the extreme value was excluded. Serum orexin-A therefore did not behave as a simple index of cellular injury.

**Table 4:** Serum orexin-A and its temporal change in relation to electrophysiological, neuroimaging, and metabolic findings

	*n*	Orexin-A 6–12 h	Orexin-A 72–96 h	Change (Δ)
Group comparisons, median [IQR]				
Amplitude-integrated EEG background				
Normal	26	209.1 [149.3–263.5]	186.1 [146.5–215.3]	−36.4 [− 84.9 to + 17.7]
Abnormal	15	176.3 [152.9–214.8]	205.4 [173.2–234.2]	+ 23.5 [− 5.8 to + 62.7]
* p value*		0.279	0.159	0.010*
Video EEG				
Normal	25	173.8 [142.8–237.4]	190.3 [156.0–220.1]	+ 6.7 [− 62.6 to + 51.0]
Abnormal	16	214.8 [174.8–285.5]	199.1 [149.8–237.8]	−13.3 [− 87.7 to + 17.2]
* p value*		0.082	0.759	0.530
Brain MRI				
Normal	29	190.3 [147.7–265.3]	193.0 [145.5–220.1]	−24.0 [− 78.1 to + 25.7]
Abnormal	11	186.4 [167.2–225.0]	205.4 [173.2–234.2]	+ 7.2 [− 24.6 to + 62.7]
* p value*		0.607	0.317	0.164
Seizures				
Absent	33	190.3 [143.0–258.0]	190.3 [153.0–220.1]	−4.7 [− 78.1 to + 38.8]
Present	8	188.8 [166.2–214.5]	210.6 [159.9–226.3]	+ 5.3 [− 32.6 to + 54.6]
* p value*		0.705	0.633	0.355
Spearman correlations, ρ (*p value*)				
Creatine kinase	40	−0.01 (0.95)	−0.22 (0.17)	−0.17 (0.28)
Lactate dehydrogenase	41	0.27 (0.08)	0.11 (0.48)	−0.04 (0.78)
C-reactive protein	41	0.15 (0.36)	−0.04 (0.81)	−0.06 (0.69)
pH	41	−0.14 (0.37)	−0.21 (0.20)	−0.05 (0.74)
Base deficit	41	0.12 (0.47)	0.13 (0.42)	0.15 (0.34)
Thompson score, day 1	41	0.15 (0.36)	0.26 (0.10)	0.11 (0.51)
Thompson score, day 2	41	0.05 (0.77)	0.36 (0.02)*	0.24 (0.13)
Thompson score, day 3	41	−0.03 (0.84)	0.22 (0.16)	0.25 (0.11)

Panel A: data are median [interquartile range]; between-group comparisons by Mann–Whitney U test. Panel B: Spearman rank correlation coefficients with p values in parentheses. Amplitude-integrated EEG, video EEG and brain MRI findings were each classified as normal or abnormal; MRI was available in 40 infants. The difference in orexin-A change by amplitude-integrated EEG background persisted after exclusion of the single high-leverage observation (*p* = 0.019) but was no longer apparent within the hypothermia group alone (p = 0.402), which contained 14 of the 15 abnormal backgrounds. Δ change from 6–12 h to 72–96 h, *EEG* electroencephalography, *h* hour, *IQR* interquartile range, *MRI* magnetic resonance imaging, *n* number. *p < 0.05

## Discussion

To our knowledge, this is the first clinical study of serum orexin-A dynamics in neonates with HIE during the early postnatal course. The principal finding was that the temporal pattern of orexin-A differed by clinical management: concentrations declined significantly in infants managed without TH but were relatively preserved, with no significant within-group change, in those who were cooled. This temporal dissociation carried a medium-to-large effect size (Cohen’s d = 0.75) and was present despite similar absolute concentrations at each time point. Higher 72–96 h concentrations also appeared to track severity, but these associations rested on high-leverage observations and did not survive rank-based analysis. Taken together, the findings suggest that orexin-A may act as a dynamic, trajectory-based marker of the evolving response to HIE.

A central implication of this study is that the clinical relevance of serum orexin-A may lie less in a single absolute value than in its temporal trajectory. Absolute concentrations at 6–12 h and 72–96 h did not distinguish the management groups (*p* = 0.078 and *p* = 0.082, respectively), nor did they consistently separate Sarnat stages or correlate with Thompson scores in outlier-insensitive analyses. Within-subject change, by contrast, differed between groups and carried a medium-to-large effect size. This change-based signal held up in a permutation test and after removal of the single extreme value, and it separated the management groups with an AUC of 0.79, a degree of discrimination that no single-time-point concentration achieved. The pattern fits emerging evidence in neonatal biomarker research that serial measurements of brain-related proteins carry more prognostic information than single assessments. Serial measurement of GFAP, neurofilament light chain (NfL), tau and UCH-L1 over the first 96 h, for example, distinguishes moderate-to-severe from no or mild MRI-defined brain injury and predicts neurodevelopmental outcome at 17–24 months, with NfL most informative at the later time point [20]. Our data suggest that orexin-A may show a similarly time-dependent profile, which would be worth formalizing in adequately powered prospective studies.

The orexinergic system is well established in the control of diurnal rhythm, feeding and energy homeostasis, arousal, emotion, locomotion and cognition [21–26]. Orexins A and B are secreted from the lateral hypothalamus, the perifornical area and the dorsomedial hypothalamus [27]; orexin-A binds OX1R and OX2R with roughly equal affinity, whereas orexin-B prefers OX2R [26]. Both receptors are expressed widely in the brain and on microglia and astrocytes [28]. A growing body of work supports neuroprotective and anti-neuroinflammatory functions: intracerebroventricular orexin-A reduces infarct area after middle cerebral artery occlusion [29], and peripherally administered orexin-A reduces demyelination, astrogliosis and microglial activation in experimental autoimmune encephalomyelitis [30]. Intranasal orexin-A accelerates EEG and behavioral recovery after cardiac-arrest asphyxia [31] and protects neurons against oxidative stress and excessive autophagy [32, 33].

Preclinical evidence for orexin-A in cerebral hypoxia–ischemia continues to expand. In ischemia–reperfusion injury, orexin-A suppresses excessive neuronal autophagy through the OX1R-mediated MAPK/ERK/mTOR pathway [16], a finding corroborated in intracerebral hemorrhage [33], and it inhibits endoplasmic reticulum stress-mediated apoptosis [34]. Most relevant to the post-asphyxial setting, orexin-A improves reperfusion in ischemic cortex by up-regulating HIF-1α and endothelial nitric oxide synthase and suppressing endothelin-1, thereby increasing cortical blood flow [35]. A role for orexin-A has also been proposed in neurodegenerative disorders such as Alzheimer’s, Parkinson’s and Huntington’s disease and amyotrophic lateral sclerosis [28, 36, 37].

Although preclinical studies support neuroprotective and anti-inflammatory effects of orexin-A in experimental cerebral hypoxia and ischemia, clinical data in newborns remain very limited. In neurologically intact newborns and infants, cerebrospinal fluid orexin-A rises during the third trimester and peaks at four months of life [38]. In cord blood, serum orexin-A is elevated in neonates born to mothers with gestational diabetes mellitus [39]; only three neonates in the present cohort had a mother with gestational diabetes, and their values did not differ from the rest. Interpretation of serum orexin-A in HIE calls for caution, because the peptide is influenced by arousal, feeding, circadian regulation and autonomic activity, and may not mirror central orexinergic activity, particularly when the blood–brain barrier is disrupted. The present associations should therefore be read as exploratory and hypothesis-generating rather than as evidence of a neuroprotective role in neonatal HIE.

The between-group difference must be read in the light of the study design. TH was applied according to established eligibility criteria rather than by randomization, so every Sarnat stage II–III neonate received cooling while the normothermia group was exclusively stage I. The two groups thus represent clinically distinct populations, and the observed difference in orexin-A reflects the inseparable effects of severity and treatment. Covariate adjustment attenuated the group effect below significance (β=+55.4, *p* = 0.086), which is consistent with that reading. Our findings therefore support the descriptive conclusion that orexin-A trajectories differed between management groups, but they do not establish that hypothermia raises serum orexin-A. This is in keeping with reports that TH alters both the relationship between severity and circulating biomarkers and their temporal kinetics [40, 41].

The apparent link between higher 72–96 h orexin-A and greater severity also warrants caution. Stage III neonates showed numerically higher concentrations and a strong correlation with the third-day Thompson score, but both were largely driven by a single neonate with an extreme value (1306.7 pg/mL, approximately 5.6-fold above the cohort median) and neither was confirmed in rank-based or outlier-excluded analyses; the stage III subgroup contained only three neonates. Whether this elevation reflects underlying pathophysiology, such as greater injury, blood–brain barrier disruption, or an exaggerated stress response, or a pre-analytical or assay-related artifact cannot be determined from the available data [25, 27].

Among the biomarkers studied in HIE, orexin-A occupies a different position. The most widely investigated candidates, including GFAP, UCH-L1, NfL and tau, are structural proteins released during cellular injury and therefore report mainly on tissue damage. Orexin-A is a signaling neuropeptide, and its serum concentration may reflect the functional state of hypothalamic orexinergic pathways rather than structural injury alone. A recent systematic review and meta-analysis of neonatal encephalopathy biomarkers found that higher interleukin-6, interleukin-8 and NSE levels at birth were associated with adverse neurodevelopmental outcomes, while concluding that no single biomarker has yet been validated for routine clinical use [42]. Seen in this context, orexin-A may offer a different view of the biological response to HIE, though considerable validation is needed before any clinical application can be considered.

S100B has the longest clinical record of any biomarker in this field, and comparison with it helps to place the present findings. This calcium-binding protein of astrocytes can be measured in cord blood, serum, urine and cerebrospinal fluid; concentrations rise within hours of an asphyxial insult, relate to the severity of encephalopathy and predict death or severe disability [13, 43]. Serial sampling has proved more informative than a single measurement: urinary S100B falls during cooling in infants who respond and remains high in those who do not [44], and early values identify the MRI pattern of injury that will be seen days later [45]. Cooling itself modifies the curve, with lower serum S100B at 48 h in cooled than in non-cooled asphyxiated infants [46]. Orexin-A resembles S100B in that its clinical meaning appears to lie in a sequence rather than in a single value, and in that hypothermia appears to alter that sequence. The two markers differ in what they report. S100B is released when astrocytes are injured and when the blood–brain barrier opens, so its concentration scales with the amount of damaged tissue and higher values indicate worse injury. Orexin-A is secreted by a small hypothalamic population as part of arousal and autonomic control, and nothing in our data ties it to tissue breakdown: it was unrelated to creatine kinase, lactate dehydrogenase, pH, base deficit, MRI abnormality and video EEG findings. Its direction of change also differs from what would be expected of an injury marker, since concentrations were maintained in the more severely affected, cooled infants and fell in those with mild encephalopathy. Taken together, the two markers may reflect different aspects of the same condition: the extent of tissue injury and the functional state of a biological system. Measuring both in the same infants would therefore be a reasonable next step.

This study has several limitations. First, the sample was modest, particularly for severe HIE, which limited power for subgroup and exploratory analyses; the adjusted and Sarnat stage III analyses should be regarded as exploratory. Second, only two sampling points were used; further measurements, including a cord sample, would add insight. Third, physiological factors that influence orexin-A, such as feeding, arousal and autonomic activity, could not be systematically controlled. Fourth, serum orexin-A may not reliably reflect central activity when the blood–brain barrier is disrupted, so paired serum and cerebrospinal fluid measurements are needed for validation. Fifth, electrophysiological and imaging findings were analyzed as normal versus abnormal, a classification that cannot capture pattern-specific differences, and abnormal findings were almost restricted to the cooled infants, so these comparisons carry the same confounding as the primary one. Sixth, magnetic resonance spectroscopy was not part of the imaging protocol, so the deep grey matter lactate/N-acetylaspartate ratio, the imaging measure most closely tied to outcome, could not be related to orexin-A. Finally, neurodevelopmental follow-up was unavailable, which precluded any assessment of prognostic value.

## Conclusions

In this prospective longitudinal cohort of neonates with HIE, serum orexin-A followed distinct early trajectories in the two clinical management groups. Absolute concentrations at either time point did not differ, whereas the change from 6–12 to 72–96 h did, declining in normothermia-managed infants and being preserved or increased in those who were cooled. Higher 72–96 h concentrations were associated with severity only in exploratory, outlier-sensitive analyses, and no association was found with video EEG findings, MRI abnormality or metabolic indices. What orexin-A may add to current practice is therefore a functional rather than a structural reading of the evolving illness, obtainable from serial rather than single samples and situated in the interval between the decision to cool and the acquisition of MRI. Whether that reading carries prognostic weight remains to be determined in larger, severity-matched cohorts with neurodevelopmental follow-up, and until such data exist orexin-A should not inform clinical decisions.

## Supplementary Information

Supplementary Material 1.

Supplementary Material 2.

## Data Availability

The datasets used and/or analyzed during the current study are available from the corresponding author on reasonable request.

## Abbreviations

ANOVA: analysis of variance
AUC: area under the receiver operating characteristic curve
EEG: electroencephalography
ELISA: enzyme-linked immunosorbent assay
GFAP: glial fibrillary acidic protein
HI: hypoxic-ischemic
HIE: hypoxic-ischemic encephalopathy
HIF-1α: hypoxia-inducible factor 1α
IQR: interquartile range
MRI: magnetic resonance imaging
NfL: neurofilament light chain
NSE: neuron-specific enolase
NT: normothermia
OX1R: orexin receptor type 1
OX2R: orexin receptor type 2
ROC: receiver operating characteristic
SD: standard deviation
TH: therapeutic hypothermia
UCH-L1: ubiquitin C-terminal hydrolase-L1
