# Supplementary material for: Temporal profile of serum orexin-A in neonatal hypoxic-ischemic encephalopathy: a prospective longitudinal study
Source: BMC Pediatr. 2026 Sep 21;26:968. doi: 10.1186/s12887-026-07727-0 (PMC13647545; doi:10.1186/s12887-026-07727-0)
Supplement: Supplementary file 1 [file 12887_2026_7727_MOESM1_ESM.docx]

**Supplementary Table S1.** Serum orexin-A at 72–96 h by Sarnat stage, with sensitivity analysis.

| **Orexin-A at 72–96 hours**  **(pg/mL)** | **Sarnat I (n=26)** | **Sarnat II (n=12)** | **Sarnat III (n=3)** | **p value** |
| --- | --- | --- | --- | --- |
| Mean ± SD | 191.4±73.1 | 196.2±40.9 | 567.7±640.8 | 0.002* |
| Median [IQR] | 187.2 [146.5-206.5] | 195.1 [173.5-224.4] | 231.0 [165.5-769.0] | 0.38 |

Sensitivity analysis: the parametric ANOVA result (p=0.002) is driven by a single high-leverage observation (1306.7 pg/mL) in the Sarnat III subgroup (n=3). Kruskal–Wallis p=0.38; Mann–Whitney (III vs. I+II) p=0.24; after exclusion of the high-leverage value, Kruskal–Wallis p=0.74. The Sarnat III finding should be regarded as exploratory. h, hour; IQR, interquartile range; K–W, Kruskal–Wallis; n, number; SD, standard deviation. *p<0.05.

**Supplementary Table S2.** Sensitivity analysis: serum orexin-A at 72–96 h vs. Thompson score.

|  | **Coefficient** | **p value** | **n** |
| --- | --- | --- | --- |
| Pearson r (full dataset) | r=0.72 | <0.001* | 41 |
| Spearman ρ (full dataset) | ρ=0.22 | 0.16 | 41 |
| Pearson r (high-leverage observations excluded) | r=0.12 | 0.46 | 40 |
| Spearman ρ (high-leverage observations excluded) | ρ=0.16 | 0.33 | 40 |

High-leverage observation refers to a single neonate with an orexin-A concentration of 1306.7 pg/mL in the Sarnat stage III group. The Pearson correlation was largely driven by this observation and was not confirmed in rank-based or outlier-excluded analyses. n, number. *p<0.05.

**Supplementary Figure S1.** Study flow diagram.
